# A High-Content, Phenotypic Screen Identifies Fluorouridine as an Inhibitor of Pyoverdine Biosynthesis and *Pseudomonas aeruginosa* Virulence

**DOI:** 10.1128/mSphere.00217-16

**Published:** 2016-08-24

**Authors:** Daniel R. Kirienko, Alexey V. Revtovich, Natalia V. Kirienko

**Affiliations:** Department of BioSciences, Rice University, Houston, Texas, USA; Swiss Federal Institute of Technology Lausanne

**Keywords:** 5-fluorouracil, 5-fluorouridine, *Caenorhabditis elegans*, *Pseudomonas aeruginosa*, pyoverdine, high-throughput screening, siderophores, virulence determinants

## Abstract

*Pseudomonas aeruginosa* is an opportunistic pathogen that causes severe health problems. Despite intensive investigation, many aspects of microbial virulence remain poorly understood. We used a high-throughput, high-content, whole-organism, phenotypic screen to identify small molecules that inhibit *P. aeruginosa* virulence in *Caenorhabditis elegans*. Approximately half of the hits were known antimicrobials. A large number of hits were nonantimicrobial bioactive compounds, including the cancer chemotherapeutic 5-fluorouracil. We determined that 5-fluorouracil both transiently inhibits bacterial growth and reduces pyoverdine biosynthesis. Pyoverdine is a siderophore that regulates the expression of several virulence determinants and is critical for pathogenesis in mammals. We show that 5-fluorouridine, a downstream metabolite of 5-fluorouracil, is responsible for inhibiting pyoverdine biosynthesis. We also show that 5-fluorouridine, in contrast to 5-fluorouracil, is a genuine antivirulence compound, with no bacteriostatic or bactericidal activity. To our knowledge, this is the first report utilizing a whole-organism screen to identify novel compounds with antivirulent properties effective against *P. aeruginosa*.

**IMPORTANCE** Despite intense research effort from scientists and the advent of the molecular age of biomedical research, many of the mechanisms that underlie pathogenesis are still understood poorly, if at all. The opportunistic human pathogen *Pseudomonas aeruginosa* causes a variety of soft tissue infections and is responsible for over 50,000 hospital-acquired infections per year. In addition, *P. aeruginosa* exhibits a striking degree of innate and acquired antimicrobial resistance, complicating treatment. It is increasingly important to understand *P. aeruginosa* virulence. In an effort to gain this information in an unbiased fashion, we used a high-throughput phenotypic screen to identify small molecules that disrupted bacterial pathogenesis and increased host survival using the model nematode *Caenorhabditis elegans*. This method led to the unexpected discovery that addition of a modified nucleotide, 5-fluorouridine, disrupted bacterial RNA metabolism and inhibited synthesis of pyoverdine, a critical toxin. Our results demonstrate that this compound specifically functions as an antivirulent.

## Abstract (precis)

Despite intense research effort from scientists and the advent of the molecular age of biomedical research, many of the mechanisms that underlie pathogenesis are still understood poorly, if at all. The opportunistic human pathogen *Pseudomonas aeruginosa* causes a variety of soft tissue infections and is responsible for over 50,000 hospital-acquired infections per year. In addition, *P. aeruginosa* exhibits a striking degree of innate and acquired antimicrobial resistance, complicating treatment. It is increasingly important to understand *P. aeruginosa* virulence. In an effort to gain this information in an unbiased fashion, we used a high-throughput phenotypic screen to identify small molecules that disrupted bacterial pathogenesis and increased host survival using the model nematode *Caenorhabditis elegans*. This method led to the unexpected discovery that addition of a modified nucleotide, 5-fluorouridine, disrupted bacterial RNA metabolism and inhibited synthesis of pyoverdine, a critical toxin. Our results demonstrate that this compound specifically functions as an antivirulent.

## INTRODUCTION

*Pseudomonas aeruginosa* is a Gram-negative opportunistic pathogen responsible for a variety of soft tissue infections. Several conditions, including cystic fibrosis, immune deficiency, and hospitalization, predispose patients to infection. This pathogen is a key example of the reemergence of clinically relevant bacterial species due to widespread acquisition of drug resistance. For example, a pandrug-resistant strain of *P. aeruginosa* in Taiwan exhibited mortality rates of ~70% (1). Rates of antimicrobial resistance and the frequency of isolation of multidrug-resistant and extensively drug-resistant strains from clinical samples are increasing across all pathogenic bacterial genera (2, 3). Meanwhile, a variety of challenges (including regulatory, commercial, intellectual property, and scientific constraints) have led to the reduction, or outright elimination, of antimicrobial discovery programs by pharmaceutical companies (4 – 6).

One method to address this problem is to identify new types of treatments such as antivirulents. These drugs would prevent bacterial pathogenesis (preferably without limiting bacterial growth or generating adaptive pressure). Identification of antivirulent compounds is generally more difficult than identification of classical antimicrobials and requires a better understanding of the molecular events underlying pathogenesis. It typically requires more complex screening regimes whose output metrics are comprised of host health measures rather than bacterial growth rates. In return, compounds are much less likely to engender and promote the acquisition and spread of resistance phenotypes. In addition, they are likely to be valuable tools for probing the complexities of host-pathogen interactions.

Recently, researchers have begun to test the viability of high-throughput, high-content, phenotypic, whole-animal screens. The model nematode *Caenorhabditis elegans* is often used as a host due to its deep characterization, experimental simplicity, and powerful genetics (7 – 9). In addition, its small size allows assay miniaturization, facilitating screening in microtiter (i.e., 96- and 384-well) plates. This also facilitates assay automation and screening of small-molecule-compound libraries to identify virulence inhibitors. For example, a pilot screen using the liquid killing assay described here identified a key role for pyoverdine in *P. aeruginosa* virulence in *C. elegans* (10). Pyoverdine, a siderophore (literally, iron carrier) secreted by *P. aeruginosa* to acquire this crucial nutrient, also regulates the expression of several other pathogenic determinants, including exotoxin A, the endoprotease PrpL, and some types of biofilm (11 – 13), and can be considered a master virulence determinant. Indeed, several studies have demonstrated that disrupting pyoverdine alone is sufficient to strongly attenuate virulence in animal models of *P. aeruginosa* infection (10, 14 – 16).

Here we report that fluorinated pyrimidines strongly attenuate *P. aeruginosa* pathogenesis, likely by compromising RNA metabolism. 5-Fluorouracil (FU) and 5-fluorouridine (FUR) were shown to limit pyoverdine production. We determined that FU also temporarily restricts bacterial growth, further limiting disease. This activity was limited to FU, as the downstream metabolites FUR and 5-fluorodeoxyuridine (FUDR) did not limit growth. Our data demonstrate that the relevant intermediate metabolite for limiting virulence factor production is FUR.

## RESULTS AND DISCUSSION

### Characterization of the *C. elegans*-*P. aeruginosa* liquid killing screen.

**FIG 1:**
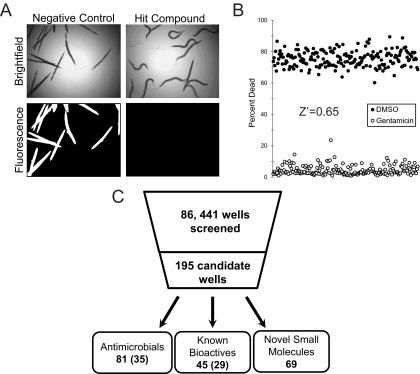
High-throughput screening of *C. elegans*-mediated liquid killing. (A) Images of worms exposed to *P. aeruginosa* and treated with either solvent control DMSO (left) or a small-molecule compound that alleviates killing (right). (B) Quantification of *C. elegans* death after treatment with *P. aeruginosa*, in the presence of either an antipseudomonal antimicrobial (gentamicin) or a vehicle control (DMSO). (C) Schematic showing the total number of wells screened, the number of hits, and the number of hit wells per category (numbers of unique structures are shown in parentheses).

In order to facilitate research on the virulence mechanisms of *P. aeruginosa* and to expedite high-throughput studies, we developed and optimized a liquid-based assay to query the pathogenesis of *P. aeruginosa* in the model nematode *C. elegans* (10). Virulence in this assay involves at least two independent determinants: the phosphatase activity of the *kinB* gene of *P. aeruginosa* (10, 17) and production of the bacterial siderophore pyoverdine (10, 18). In brief, 20 young adult *C. elegans* worms are added to each well of a 384-well plate containing *P. aeruginosa* and incubated at 25°C for approximately 36 to 40 h. Bacteria were washed away, and dead worms were stained with a membrane-impermeable dye. Survival was scored using automated image collection and analysis to remove observer bias and increase analysis throughput (Fig. 1A).

Prior to carrying out the screen, we established the Z′ factor (19) for the assay, 0.65 (Fig. 1B), which suggested that the assay has good power to identify both strong and weak hits. We also ruled out the existence of edge effects by comparing the proportions of the dead worms in the wells at the edge, in the wells in the center, and in all wells (see Fig. S1 in the supplemental material) (*P* value, 0.402).

10.1128/mSphere.00217-16.1 Figure S1  Liquid killing assay does not have edge effect. (A) Schematic of a 384-well plate showing wells used to calculate pathogenesis for edge (light gray), middle (dark gray), and all (whole plate) wells. (B) Quantified levels of *P. aeruginosa*-mediated liquid killing for each region of a representative 384-well plate. No statistically significant differences were noted based on Student’s *t* test. Error bars represent standard deviation. Three biological replicates were performed. Data from a representative replicate are shown. Download Figure S1, PDF file, 0.3 MB. Copyright © 2016 Kirienko et al. 2016 Kirienko et al. This content is distributed under the terms of the Creative Commons Attribution 4.0 International license.

### Compounds identified in the liquid killing screen include novel antimicrobials.

The liquid killing assay was used to screen 251 plates from various commercially supplied chemical diversity collections. Each plate contained between 320 and 352 compounds (the remaining wells were dedicated to positive and negative controls). Combined, these plates comprised 86,441 wells containing test compounds, of which 195 were considered hits (0.226% total rate, similar to the rates determined in previous *C. elegans* phenotypic screens) (8) (Fig. 1C). Initial analysis showed that 81 of these hits represent known antimicrobials. We identified a total of 35 different compounds in this group; each was independently found 1 to 5 times in the screen, and some compounds were present in two or more plates. Most of the antimicrobials identified belong to the fluoroquinolone and tetracycline classes of antibiotics (Table 1). A total of 45 wells with hits contained known bioactive molecules, with 29 different structures identified. The 69 remaining molecules fall into at least three nonexclusive classes: novel antimicrobials (compounds that limit bacterial growth or kill bacteria), antivirulents (compounds that inhibit bacterial pathogenesis without affecting growth), and host immune stimulators (compounds that predominantly act by promoting host survival and have little to no impact on the pathogen). All three categories may lead to the discovery of useful molecules with roles ranging from probe compounds to potential leads for therapeutic development.

**TABLE 1:** Classes of known antimicrobials discovered in high-throughput screen

Name	Occurrence^a^
Fluoroquinolones	
Ciprofloxacin	2
Clinafloxacin	2
Difloxacin	1
Enrofloxacin	2
Fleroxacin	1
Flumequine	1
Gatifloxacin	4
Gemifloxacin	2
Levofloxacin	4
Lomefloxacin	5
Moxifloxacin	3
Nadifloxacin	1
Norfloxacin	3
Ofloxacin	3
Pazufloxacin	2
Pefloxacin	5
Prulifloxacin	1
Rufloxacin	2
Sarafloxacin	3
Sparfloxacin	1
Tosufloxacin	2
Trovafloxacin	1
	
Tetracyclines	
Chlortetracycline	2
Demeclocycline	4
Doxycycline	4
Meclocycline	3
Minocycline	5
Oxytetracycline	3
Tetracycline	1
	
Other	
Alexidine	1
Dirithromycin	1
Polymyxin B	2
Rifabutin	1
Thiostrepton	2
Tobramycin	1

a Occurrence, number of times the compound appeared in the screen.

Of the 69 novel small molecules, 60 were commercially available and were purchased as powders. To limit vendor misidentification, compounds were purchased from a second vendor when possible (approximately half of the compounds), and compound identification verification (by, e.g., nuclear magnetic resonance [NMR], high-pressure liquid chromatography [HPLC], etc.) was requested from all vendors. Hits were resuspended in dimethyl sulfoxide (DMSO) at a concentration of 5 µg·µl^−1^ and retested in the *C. elegans* assay. Of the 60 commercially available hits, 56 (93.3%) passed this validation step.

These 56 compounds were subjected to a variety of tests for preliminary characterization. First, MIC values for each compound were determined and compared to effective rescue concentration (EC, defined as the minimum concentration required for statistically significant rescue of *C. elegans*). Two hits, LK10 (3-hydrazinoquinoxaline-2-thiol) and LK55, showed MICs less than twice the EC. On this basis, they were classified as antimicrobials (i.e., traditional antibiotics that limit bacterial growth). Decoding of LK55 and LK59 (the latter of which was not commercially available for retesting) revealed that they are close analogs of the fluoroquinolone antimicrobial ofloxacin (PubChemID 4583; see Fig. S2 in the supplemental material). On this basis, we consider them likely antibiotics and have deprioritized their further study.

10.1128/mSphere.00217-16.2 Figure S2  Previously uncharacterized antimicrobials were identified in a high-throughput screen. Structures, names, and IUPAC identifiers for the known antimicrobial ofloxacin (A), and its analogs LK55 (B) and LK59 (C). LK10 (D) also possesses antimicrobial activity. Download Figure S2, PDF file, 0.5 MB. Copyright © 2016 Kirienko et al. 2016 Kirienko et al. This content is distributed under the terms of the Creative Commons Attribution 4.0 International license.

LK10 has previously been shown to possess strong antimicrobial activity against *Helicobacter pylori* (20). Neither literature review nor bacterial growth assays suggested than any of the other 54 novel small molecules ordered are likely to function as antimicrobials. Our current hypothesis is that they are novel antivirulents (i.e., virulence-blocking compounds that do not interfere with bacterial growth) or else they promote host survival by targeting the host (e.g., by stimulating tolerance or immunity).

### 5-Fluorouracil has potent activity against *P. aeruginosa*-mediated killing of *C. elegans.*

To prioritize compounds for in-depth analysis, we decided to initially focus our attention on the 29 bioactive compounds. These belonged to three general categories, including surface decontaminants (such as hexachlorophene, phenylmercuric acetate, and thiomerosal), metal-chelating compounds (such as ciclopirox, piroctone, and nitroxamine), and fluoropyrimidines and their derivatives. Compounds in the first category most likely limit virulence by killing the pathogen and were therefore deprioritized for further study. We have previously reported the identification of ciclopirox, which led to identification of pyoverdine as the most relevant virulence factor in liquid-based killing (10). The other metal-chelating compounds may function analogously.

**FIG 2:**
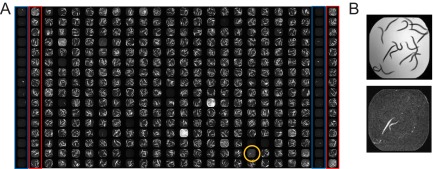
5-Fluorouracil rescues *C. elegans* from *P. aeruginosa*-mediated killing. (A) Tiled fluorescence images of a plate from NINDS custom collection 2. A well (O18) containing 5-fluorouracil is circled in orange. Columns 1 and 23 represent gentamicin (positive control; outlined in blue). Columns 2 and 24 represent DMSO (negative control; outlined in red). Columns 3 to 22 represent 320 different small molecules, the majority of which do not alleviate host killing. Library plates were screened in duplicate. Data from a representative replicate of the plate noted are shown. (B) Single-well images of worms exposed to 5-fluorouracil (FU; well O18) showing bright-field results (top) and fluorescence (bottom).

The fluoropyrimidines included carmofur and FU, which was identified as a hit on 5 different plates (representative data are shown in Fig. 2). We chose FU for further characterization because it showed the strongest activity of any of the compounds for which dose-response analysis was performed. In addition, it has the advantage of being a well-studied molecule with a thoroughly mapped metabolism.

FU was purchased as a powder, dissolved, and retested to verify that it rescued liquid killing. Transcriptome profiling of worms treated with 50 µM FU showed no differentially regulated genes compared to solvent controls (see Fig. S3 in the supplemental material). While initially surprising, this result is consistent with the biology of postmitotic, sterile *C. elegans*. In brief, young adult *C. elegans* nematodes are comprised of two tissue types: postreplicative, terminally differentiated somatic cells and actively dividing germline cells. However, the *C. elegans* strain we used has a mutation in the *glp-4* gene that abolishes germline cells at an early stage of development. As such, the worms used in the assay were entirely postmitotic. This would preclude the cell cycle disruption by FU. This, combined with the observation that no genes were differentially regulated, suggests that it is highly unlikely that FU is acting upon the host.

10.1128/mSphere.00217-16.3 Figure S3  Zero host genes are differentially expressed due to 5-fluorouracil. Microarray transcriptional profiling of worms exposed to either solvent (dimethyl sulfoxide) control (*x* axis) or 5-fluorouracil (50 mM, *y* axis). Each *C. elegans* probe set is represented by a single circle. Both Student’s *t* test and a modified Wilcoxon rank test returned zero genes that were differentially expressed (see Materials and Methods for criteria). Three microarray *C. elegans* GeneChips were used for each condition. The median value for each probe was used for plotting. Download Figure S3, PDF file, 0.3 MB. Copyright © 2016 Kirienko et al. 2016 Kirienko et al. This content is distributed under the terms of the Creative Commons Attribution 4.0 International license.

**FIG 3:**
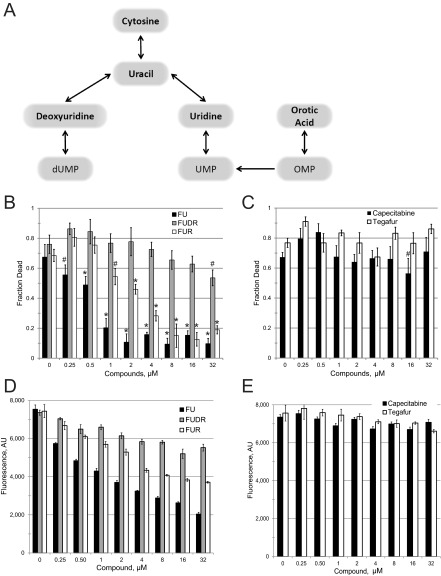
5-Fluorouracil-based chemotherapeutics interfere with pyoverdine biosynthesis. (A) Schematic of 5-fluorouracil metabolism in *P. aeruginosa*. The diagram shows the complex relationships between pyrimidine analogs. OMP, orotate monophosphate. Compounds tested with 5-fluorinated derivatives are indicated in bold. (B and D) Dose-response curves of *C. elegans* exposed to *P. aeruginosa* under liquid killing conditions in the presence of 5-fluorouracil (FU; black), 5-fluorodeoxyuridine (FUDR; gray), or 5-fluorouridine (FUR; white). Killing results (B) and pyoverdine fluorescence after 24 h of infection (D) are shown. (C and E) Dose-response curves of *C. elegans* exposed to *P. aeruginosa* in the presence of capecitabine (black) or tegafur (white). Killing results (C) and pyoverdine fluorescence after 24 h of infection (E) are shown. Statistical significance: *, *P* < 0.01; #, *P* < 0.05 (based on Student’s *t* test). At least three biological replicates were done for each compound. Data from a representative replicate are shown.

Since both carmofur and FU were identified as hits, we purchased and tested other fluorinated pyrimidines, including FUR and FUDR, which are downstream metabolites of FU (Fig. 3A) (21). We also tested two FU prodrugs, tegafur and capecitabine, which are metabolized to FU in humans (22, 23). FUR, but not FUDR, showed a significant ability to inhibit *C. elegans* killing at low micromolar concentrations (Fig. 3B). Neither capecitabine nor tegafur showed any significant ability to rescue worm killing (Fig. 3C), suggesting that the absorption and/or metabolism of these drugs to FU is inefficient in *P. aeruginosa* and/or *C. elegans*. Supplementation with nonfluorinated uracil also had no discernible effect on pathogenesis at the concentrations tested for fluorinated derivatives (see Fig. S4 in the supplemental material), indicating that these compounds are not merely affecting cellular uracil flux.

10.1128/mSphere.00217-16.4 Figure S4  Uracil supplementation does not affect *C. elegans* survival after *P. aeruginosa* treatment. *C. elegans* death after exposure to *P. aeruginosa* in the presence of various concentrations of uracil. No statistically significant differences were noted based on Student’s *t* test. Error bars represent standard errors of the means (SEM). Two biological replicates were performed. Data from a representative replicate are shown. Download Figure S4, PDF file, 0.3 MB. Copyright © 2016 Kirienko et al. 2016 Kirienko et al. This content is distributed under the terms of the Creative Commons Attribution 4.0 International license.

**FIG 4:**
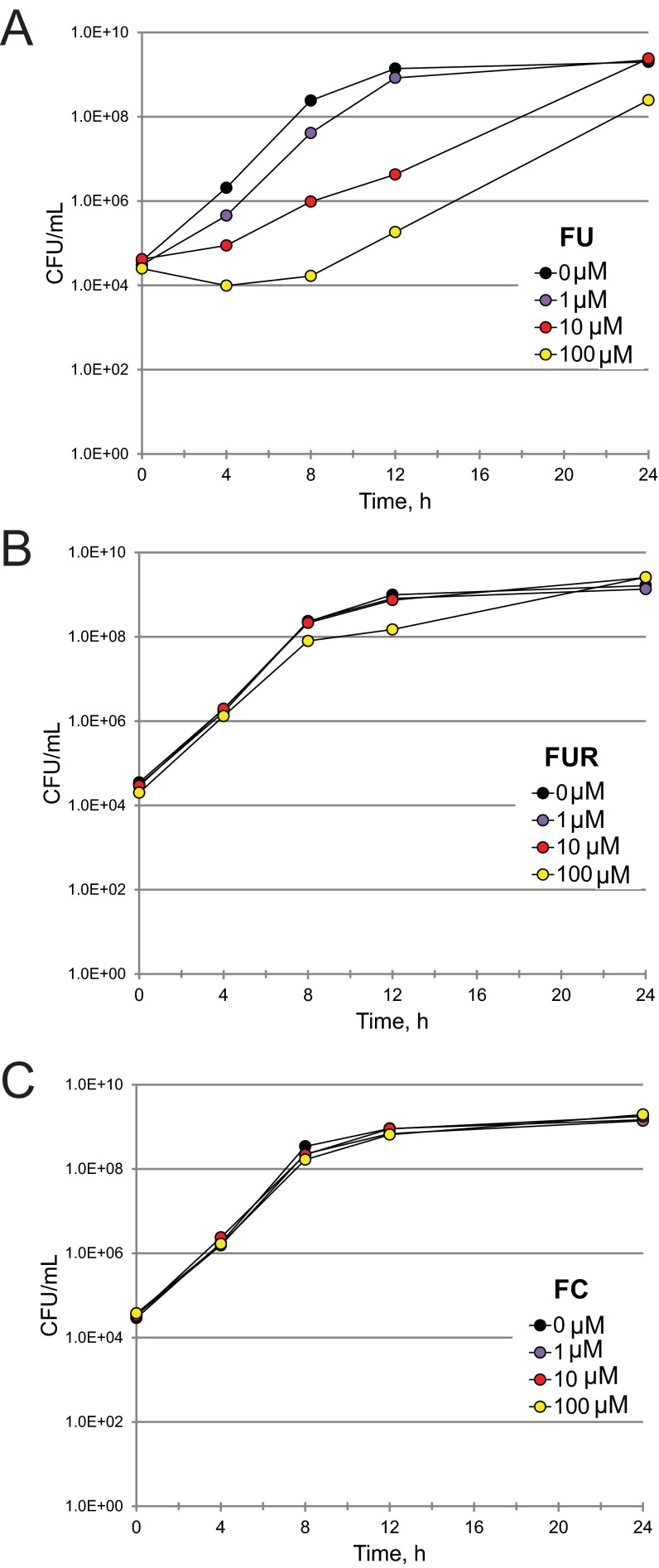
5-Fluorouracil acts as a bacteriostatic agent, but 5-fluorouridine and 5-fluorocytosine have no effect on bacterial growth. *P. aeruginosa* was exposed to various concentrations of 5-fluorouracil (FU) (A), 5-fluorouridine (FUR) (B), or 5-fluorocytosine (FC) (C) for various times. Bacteria were then serially diluted to calculate the number of viable CFU per milliliter of medium. At least three biological replicates were done for each compound. Data from a representative replicate are shown.

As previously noted, these compounds may be functioning as antimicrobials, virulence inhibitors, or both. FU and FUR showed MIC values more than 2 orders of magnitude larger than their EC values, making it quite unlikely that they act as classic antimicrobials (Table 2). However, MICs are determined on the basis of an endpoint assay and are insensitive to bacterial viability. Therefore, we also quantified the bacterial titer of *P. aeruginosa* treated with FU and FUR by serial dilution (Fig. 4A and B). Although the MIC assay suggested that FU lacked significant antimicrobial activity, the CFU assay revealed a clear, but temporary, bacteriostatic effect when *P. aeruginosa* was treated with FU. We hypothesize that the transience of this effect is a consequence of *P. aeruginosa* activating multisubstrate *mexAB* efflux pumps when under biological stress (24). FUR had no discernible effect on bacterial growth even at the highest concentration tested, which was more than 40-fold higher than the EC.

**TABLE 2:** EC and MIC data for fluorinated pyrimidine analogs

Analog	EC (µM)	MIC (µM)	
		SK medium	LB medium
FU	0.375	300	400
FUR	2.437	400	>400
FC	21.2	>400	>400

### 5-Fluorouridine is a potent antivirulent that prevents pyoverdine biosynthesis.

A previous report by Imperi and colleagues demonstrated that 5-fluorocytosine (FC), a fluoropyrimidine that certain organisms can efficiently convert to FU, limits pyoverdine production. The identified mechanism involved reducing transcription of the sigma factor *pvdS*, which is responsible for pyoverdine biosynthesis (15). Interestingly, Imperi et al. illustrated that conversion of FC to FU is required for virulence attenuation. However, Imperi et al. did not test metabolites downstream of FU to evaluate their impact on *pvdS*, nor was a molecular mechanism identified.

**FIG 5:**
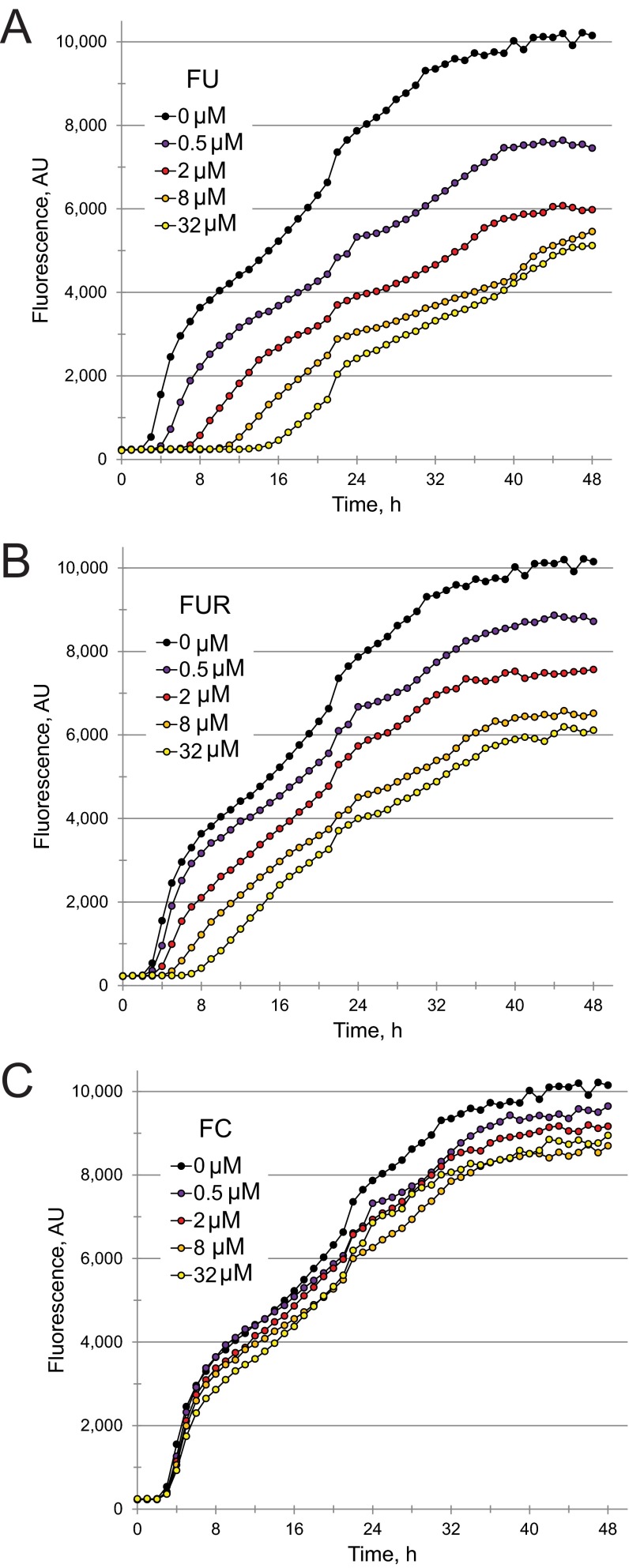
5-Fluorouracil and 5-fluorouridine inhibit pyoverdine fluorescence. (A to C) Time-lapse measurement of pyoverdine production by *P. aeruginosa* grown under liquid killing conditions in the presence of 5-fluorouracil (FU) (A), 5-fluorouridine (FUR) (B), or 5-fluorocytosine (FC) (C). At least three biological replicates were done for each compound. Data from a representative replicate are shown.

We hypothesized that conversion of FU (and FC, by extension) to FUR is responsible for attenuating pyoverdine production. Therefore, we measured pyoverdine fluorescence in *P. aeruginosa* exposed to various concentrations of FU, FUR, FUDR, tegafur, or capecitabine (Fig. 3D and E and 5A and B). Our results correlated with the virulence observations: FU and FUR inhibited pyoverdine production, inhibition by FUDR was weak (even at the highest concentration tested), and neither tegafur nor capecitabine had any significant effect. We also tested whether FC inhibited bacterial growth (Fig. 4C), pyoverdine production (Fig. 5C), and *P. aeruginosa*-mediated *C. elegans* pathogenesis (Table 2). We saw no significant effect of FC treatment on bacterial growth kinetics, even at concentrations as high as 100 µM. While we observed an effect on pyoverdine from FC treatment, it was more modest than that seen with FU or FUR under our test conditions (Fig. 5). This was consistent with our measurements of their relative EC values (Table 2).

To further test our hypothesis that FUR was necessary for virulence attenuation, we took advantage of an alternative *P. aeruginosa* pathway for producing UMP. This alternate pathway normally converts orotate into UMP through orotate monophosphate. This biochemical pathway entirely bypasses uracil (Fig. 3A). Similarly, addition of 5-fluoroorotate (FO) should lead to the production of FUR (with few to no other uracil by-products). Although FO was generally less effective than FUR, we observed significant decreases in host mortality and pyoverdine production when FO was added (see Fig. S5 in the supplemental material). The decreased effect is likely to result from incomplete conversion of FO into FUR, limiting cellular FUR concentrations.

10.1128/mSphere.00217-16.5 Figure S5  5-Fluoroorotate is capable of increasing host survival and decreasing pyoverdine biosynthesis. (A) Survival of *C. elegans* exposed to *P. aeruginosa* in the presence of various concentrations of 5-fluoroorotate (FO). (B) Time-lapse measurement of pyoverdine production by *P. aeruginosa* in the liquid killing assay, in the presence of 32 µM FO. FUR is shown as a control in panels A and B. Statistical significance: *, *P* < 0.01; #, *P* < 0.05 (based on Student’s *t* test). Error bars in panels A and B represent SEM. Two biological replicates were performed for panels A and B. Data from a representative replicate are shown. Download Figure S5, PDF file, 0.4 MB. Copyright © 2016 Kirienko et al. 2016 Kirienko et al. This content is distributed under the terms of the Creative Commons Attribution 4.0 International license.

To rule out the possibility that FU was merely preventing the maturation or secretion of pyoverdine, we collected bacteria (grown in the presence or absence of FU) via centrifugation and then boiled the bacteria. This would release any unexported, fluorescent pyoverdine, which would be stable under these conditions (data not shown). The amount of pyoverdine released in this fashion was virtually undetectable (see Fig. S6 in the supplemental material), ruling out the possibility that FU prevents maturation or secretion rather than synthesis.

10.1128/mSphere.00217-16.6 Figure S6  FUR does not affect pyoverdine secretion. Paired samples of cultures were grown in the presence (32 µM) or absence of FUR. Bacteria were pelleted from one sample from each pair, and the supernatant was retained. Bacteria were resuspended in equal volumes of S basal minimal medium. Pyoverdine fluorescence was measured for each sample. Subsequently, samples were boiled for 15 min, and pyoverdine fluorescence was measured once more. Pyoverdine fluorescence levels are indicated in arbitrary units. Two biological replicates were performed; data from a representative replicate are shown. Error bars represent SEM. Download Figure S6, PDF file, 0.3 MB. Copyright © 2016 Kirienko et al. 2016 Kirienko et al. This content is distributed under the terms of the Creative Commons Attribution 4.0 International license.

**FIG 6:**
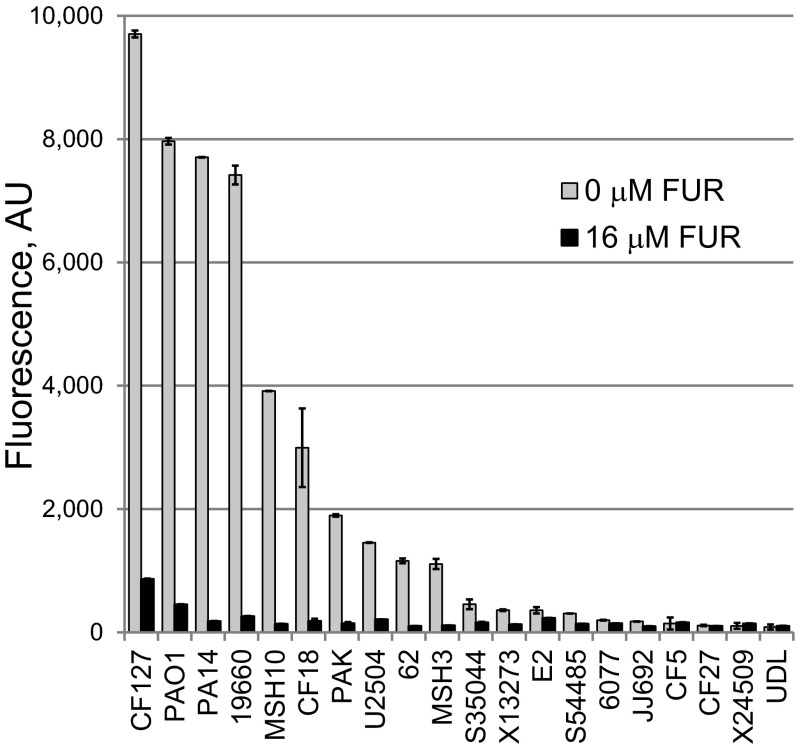
5-Fluorouridine efficiently inhibits pyoverdine fluorescence across a variety of *P. aeruginosa* isolates. Data represent pyoverdine fluorescence after 20 h of bacterial growth with and without 16 µM FUR (liquid killing medium, 28°C). Strains are ordered according to their level of pyoverdine production in the absence of FUR. Error bars represent standard errors of the means (SEM). Two biological replicates were performed. Data from a representative replicate are shown.

Pyoverdine structures show sufficient variation between pseudomonads to allow them to function as taxonomic indicators (25 – 27). Although the machinery involved in pyoverdine biosynthesis is the same, the amino acid chains differ significantly between strains. Therefore, we tested 19 additional strains of *P. aeruginosa* (28), including strains from a variety of infection sites (sepsis, ocular, burn, etc.) and from several environmental samples (Fig. 6). Although not all of the strains synthesized high levels of pyoverdine, FUR strongly inhibited pyoverdine biosynthesis in all of the strains that did.

The available data suggest a model wherein FUR (and, by extension, FU and FC) limits virulence by inhibiting pyoverdine production. The fact that FUDR shows very little activity suggests that FU does not limit pyoverdine production by disrupting thymidylate synthase. Instead, the likeliest explanation is that FUR is phosphorylated and incorporated into cellular RNA pools in place of normal UTP. Extensive biochemical studies from the 1960s and 1970s have shown that fluorinated uracil is effectively incorporated into ribosomal, transfer, and mRNA pools, with pleiotropic effects. For example, incorporation into rRNA disrupts maturation and prevents processing of the 45S and 38S precursor rRNAs in eukaryotes (29, 30). rRNA processing is also compromised in bacteria (31). FUR incorporation into tRNAs retards aminoacylation of glutamate, aspartate, glutamine, histidine, and especially lysine tRNAs (32). These tRNAs also bind to ribosomes less efficiently and compromise overall protein production (32). Finally, incorporation into mRNAs results in unpredictable, low-frequency translational errors when FUR in the transcript is misread as a cytosine rather than as a uracil (33).

### Antivirulent effects of 5-fluorouridine are specific to pyoverdine.

**FIG 7:**
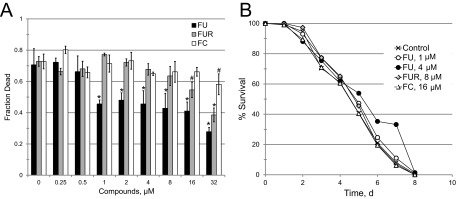
Amelioration of killing by 5-fluorouridine is specific to pyoverdine-mediated pathology. (A) Dose-response curve of *C. elegans* killing mediated by the *P. aeruginosa* PA14 *pvdA* mutant (which is defective in pyoverdine biosynthesis) after exposure to various concentrations of 5-fluorouracil (FU), 5-fluorouridine (FUR), or 5-fluorocytosine (FC). (B) *C. elegans* survival during a lethal, plate-based, *P. aeruginosa* infection assay. Plates were supplemented with FU, FUR, or FC at the concentrations shown. Pyoverdine was dispensable for killing in this assay. *, *P* < 0.01; #, *P* < 0.05 (based on Student’s *t* test) (A); *P* < 0.05 (for 4 µM FU, based on log-rank test [http://bioinf.wehi.edu.au/software/russell/logrank/]) (B). Three (A) or two (B) biological replicates were done for each experiment. Data from representative replicates are shown. d, day.

To verify that the relevant virulence factor being disrupted by FU, FUR, and FC is pyoverdine, we tested whether these compounds could rescue pathogenesis induced by strains of *P. aeruginosa* with transposons inserted in pyoverdine biosynthesis genes (34) such as *pvdA* (Fig. 7A). While the pathogenicity of these strains is strongly reduced compared to wild-type levels, they still retain significant virulence (10). In comparison to wild-type *P. aeruginosa*, treatment of *pvdA* mutants with FUR or FC showed a decreased ability to rescue worms. Although FU efficacy was strongly diminished, significant rescuing activity was retained (compare Fig. 2A and 7A). This is likely due to the bifunctional effects of FU on both pyoverdine production and bacterial growth; only the former would be affected by disrupting pyoverdine biosynthesis.

We also tested whether FU, FUR, or FC could rescue *C. elegans* infection with *P. aeruginosa* on solid media (also known as slow-kill assays), where pyoverdine is dispensable for pathogenesis. None of the fluoropyrmidines tested exhibited any attenuation at concentrations close to EC values for liquid killing (Fig. 7B). Consistent with other data, 4 µM FU showed a weak effect (*P* < 0.05). This was near the concentration where the bacteriostatic effect of FU becomes apparent. These results further bolster the conclusion that the primary effect of the compounds is to limit pyoverdine toxicity.

Ueda et al. (35) have shown that a number of pathogenesis pathways are compromised by treatment with FU, including biofilm formation, swarming, and production of elastase, pyocyanin, rhamnolipids, and at least one quorum sensing signal. It is worth noting that virtually all of these virulence factors have been linked to pyoverdine activity (12), suggesting that FU’s ability to compromise pyoverdine may underlie its ability to disrupt all of the others as well. Further work will be necessary to address this issue.

Although our data suggest the potential utility of FU and FUR as antivirulents, the generally severe side effects of fluorinated pyrimidines preclude their widespread use as either antimicrobials or antivirulents in any but the most serious of situations (infection with a pandrug-resistant strain of *P. aeruginosa*, for example). Available data for FUR suggest that it exhibits gastrointestinal toxicity comparable to that of FU (36), but our data in *C. elegans* suggest that antivirulent doses of FUR exhibit relatively mild toxicity (see Fig. S7 in the supplemental material). Further work remains to be done to clarify this matter. Regardless of their value as antivirulents in humans, fluorinated pyrimidines will remain useful as tool compounds for studying the regulation of pyoverdine production. This also highlights the utility of using unbiased approaches to gain insights into host-pathogen interactions.

10.1128/mSphere.00217-16.7 Figure S7  Prolonged exposure to 5-fluorouridine is not toxic to *C. elegans*. Toxicity of FUR or a solvent control was assayed at different time points and concentrations under liquid killing conditions. *E. coli* strain OP50 was substituted for *P. aeruginosa*. No statistically significant increases in host death were noted based on Student’s *t* test results. Error bars represent SEM. Three biological replicates were performed, and data from a representative replicate are shown. Download Figure S7, PDF file, 0.3 MB. Copyright © 2016 Kirienko et al. 2016 Kirienko et al. This content is distributed under the terms of the Creative Commons Attribution 4.0 International license.

## MATERIALS AND METHODS

### Strains.

The SS104 [*glp-4*(*bn2*)] *C. elegans* strain was maintained on nematode growth medium (NGM) seeded with *Escherichia coli* strain OP50 at 15°C (37). For all experiments using wild-type *P. aeruginosa*, we used *P. aeruginosa* strain PA14, a clinical isolate described elsewhere (38). The PA14 *pvdA* mutant has a Mariner transposon inserted into the *pvdA* locus (34) and was sequenced prior to use. The other 19 strains of *P. aeruginosa* tested have been described previously (28).

### Media.

NGM (standard nematode growth medium), S basal minimal medium, LK medium (modified liquid NGM used for liquid killing assays with *P. aeruginosa*), and SK modified NGM used for plate-based infection with *P. aeruginosa* are all described elsewhere (39).

### Liquid killing assay.

The liquid killing assay was performed as previously described (39, 40). Z′ factor determinations were performed in 384-well plates, where half of the wells contained DMSO as a negative control and half contained 100 µg·ml^−1^ gentamicin as a positive control. Unbiased discrimination between living and dead worms was performed on the basis of staining by the cell-impermeant fluorescent dye Sytox Orange (Invitrogen, Carlsbad, CA) (8). Cell Profiler software (http://cellprofiler.org/) was used for quantitative image processing and determination of worm mortality (41, 42).

High-throughput chemical screening was performed at the National Screening Laboratory for the Regional Centers of Excellence in Biodefense and Emerging Infectious Diseases (NSRB) at Harvard Medical School. Compounds were screened in duplicate at a final concentration of approximately 20 µg·ml^−1^. Each 384-well plate contained at least 30 wells dedicated to solvent (DMSO) controls. Compounds were considered primary hits if survival in both replicates was >3 standard deviations (SD) above the mean value for negative controls on the same plate.

EC values were determined in the liquid killing assay as the lowest compound concentration resulting in statistically significant rescue of worms (*P* < 0.05 [based on Student’s *t* test]). EC values for each compound represent an average of at least 4 biological replicates, with at least 4 wells per replicate.

Pyoverdine production under liquid killing conditions was measured spectrophotometrically (excitation wavelength [Ex], 405 nm; emission wavelength [Em], 460 nm) using a Cytation5 multimode plate reader/imager (Molecular Devices, Sunnyvale, CA).

### MIC assay.

To determine the MIC of compounds for preventing bacterial growth, *P. aeruginosa* strain PA14 was grown in standard LB overnight and diluted 100,000-fold in either LB or in SK media. Compounds were 2-fold serially diluted and mixed with bacteria 1:1. Growth inhibition was visually scored on the basis of turbidity. Two wells were used per condition, and at least three biological replicates were performed.

### CFU assay.

To determine the number of CFU, aliquots were taken at appropriate time points and serially diluted 5-fold in water prior to plating onto solid LB plates (2% agar). Colonies were counted under a dissecting microscope.

### RNA extraction and microarray analysis.

Young adult worms (10,000) were plated onto 10-cm NGM plates supplemented with either DMSO or 50 µM FU. After 8 h, worms were washed into a 15-ml conical tube with S basal medium and rinsed twice. Afterward, worms were resuspended in TRI reagent (MRC, Inc., Cincinnati, OH) and frozen at −80°C. After thawing, RNA was extracted according to manufacturer’s protocols and purified further using RNeasy columns (Qiagen, Gaithersburg, MD). cRNA samples were prepared and hybridized to full-genome GeneChips for *C. elegans* (GPL200; Affymetrix, Santa Clara, CA) according to manufacturer’s protocols. Three biological replicates were tested for each condition. Gene expression was analyzed using GCRMA (http://www.bioconductor.org). Differentially regulated genes were determined as previously described (43). For nonparametric determination of significantly affected genes, the following values were chosen: fold change, >2; modified Wilcoxon rank coefficient, >1.5-fold; absolute expression level, >80 arbitrary units (AU). For the determination of significantly affected genes using the assumption of normal distribution, MAS Suite and following criteria were used: fold change, >2; Student’s *t* test value, <0.01 (after multiple sample correction); and Absent/Present calls of MAS suite.

### Slow-killing assay.

The slow-killing assay was performed as previously described (39). At least two biological replicates were performed for each experiment. Each biological replicate consisted of three plates with 50 worms per plate. Worms that left the surface of the agar were eliminated from the scoring.

### Accession number(s).

Microarray data have been deposited in the NIH Gene Expression Omnibus database under accession number GSE85342.
