# Supplementary figures and images for: A High-Content, Phenotypic Screen Identifies Fluorouridine as an Inhibitor of Pyoverdine Biosynthesis and Pseudomonas aeruginosa Virulence
Source: mSphere. 2016 Aug 24;1(4):e00217-16. doi: 10.1128/mSphere.00217-16 (PMC4999921; doi:10.1128/mSphere.00217-16)

A

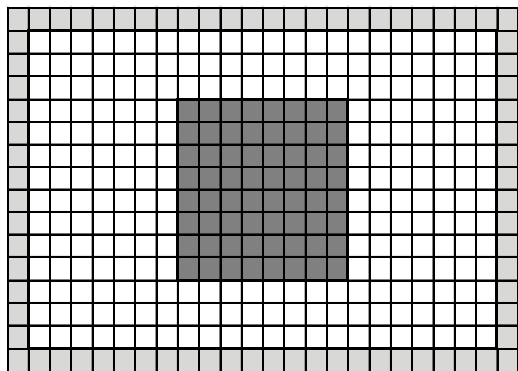

B

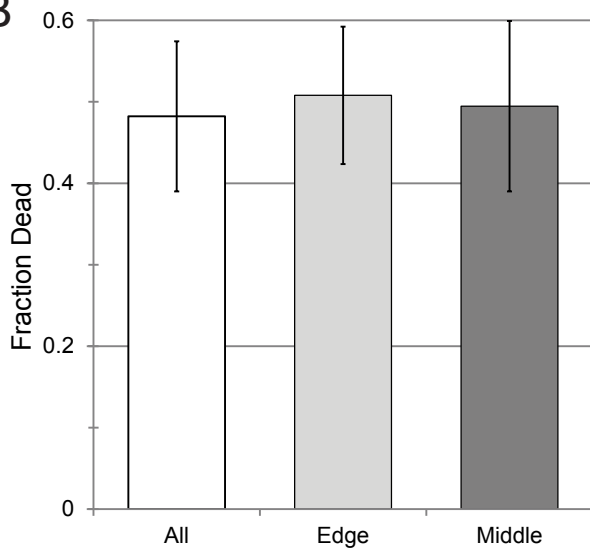

Figure S1

Supplement: Figure S1 [file sph004162135sf1.pdf]

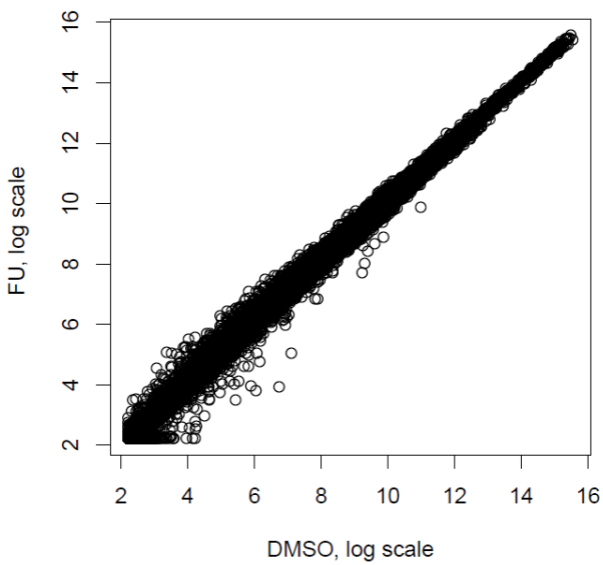

Figure S3

Supplement: Figure S3 [file sph004162135sf3.pdf]

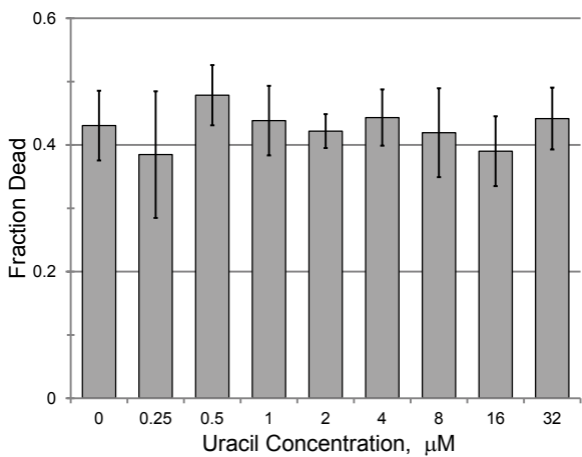

Figure S4

Supplement: Figure S4 [file sph004162135sf4.pdf]

A

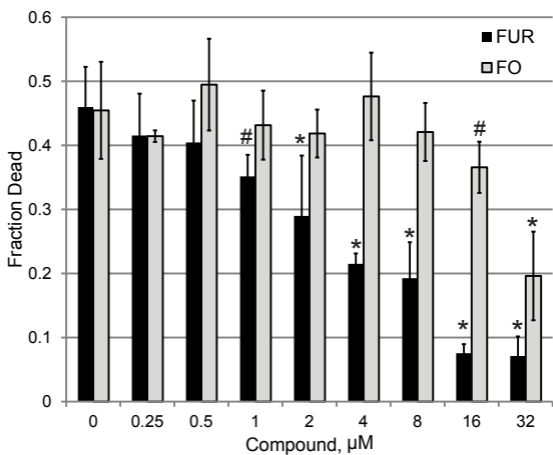

B

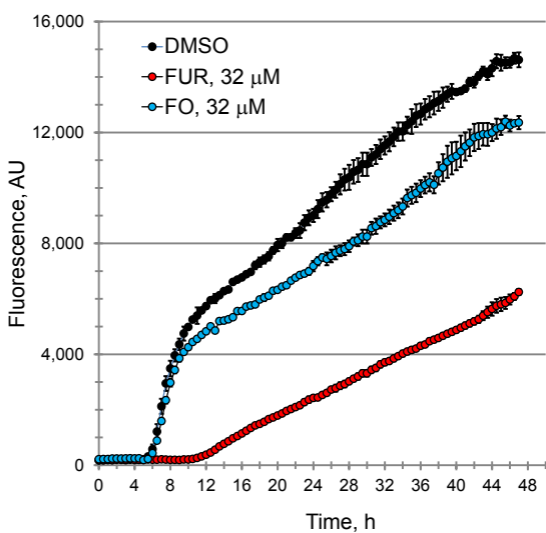

Figure S5

Supplement: Figure S5 [file sph004162135sf5.pdf]

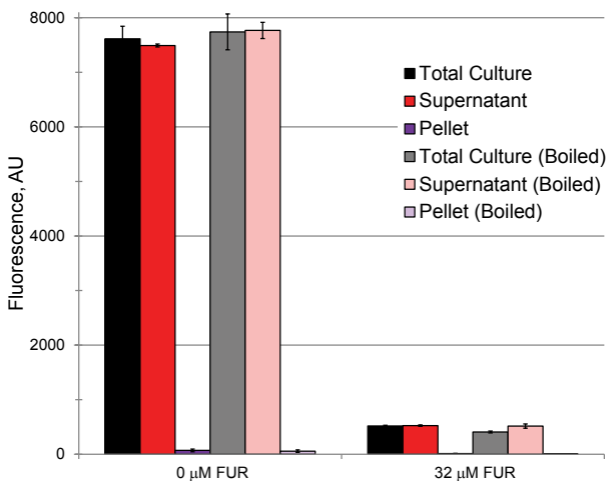

Figure S6

Supplement: Figure S6 [file sph004162135sf6.pdf]

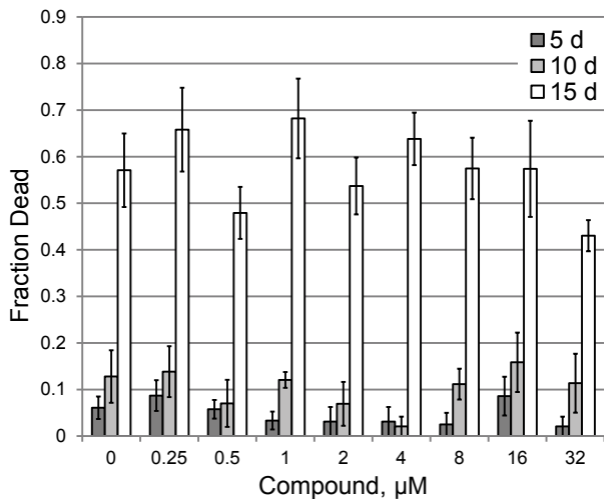

Figure S7

Supplement: Figure S7 [file sph004162135sf7.pdf]
