# Supplementary material for: A High-Content, Phenotypic Screen Identifies Fluorouridine as an Inhibitor of Pyoverdine Biosynthesis and Pseudomonas aeruginosa Virulence
Source: mSphere. 2016 Aug 24;1(4):e00217-16. doi: 10.1128/mSphere.00217-16 (PMC4999921; doi:10.1128/mSphere.00217-16)
Supplement: Figure S2 [file sph004162135sf2.pdf]

A

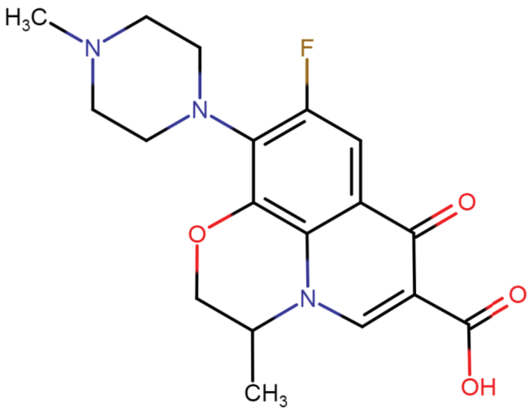

Ofloxacin

B

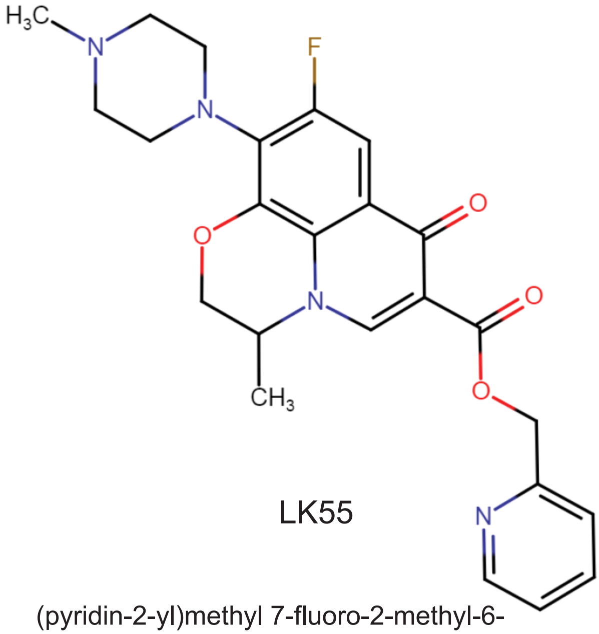

LK55

(pyridin-2-yl)methyl 7-fluoro-2-methyl-6-(4-methylpiperazin-1-yl)-10-oxo-4-oxa-1-azatricyclo[7.3.1.$0^{5,13}$]trideca-5(13),6,8,11-tetraene-11-carboxylate

C

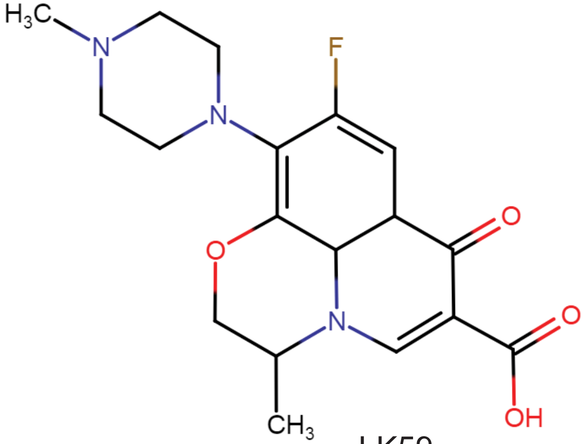

LK59

7-fluoro-2-methyl-6-(4-methylpiperazin-1-yl)-10-oxo-4-oxa-1-azatricyclo[7.3.1.$0^{5,13}$]trideca-5,7,11-triene-11-carboxylic acid

D

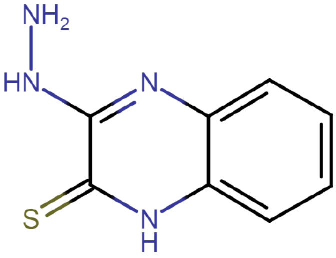

LK10

3-hydrazinoquinoxaline-2-thiol

Figure S2
